# Flexible Signaling of Myeloid C-Type Lectin Receptors in Immunity and Inflammation

**DOI:** 10.3389/fimmu.2018.00804

**Published:** 2018-04-26

**Authors:** Carlos del Fresno, Salvador Iborra, Paula Saz-Leal, María Martínez-López, David Sancho

**Affiliations:** ^1^Immunobiology Laboratory, Centro Nacional de Investigaciones Cardiovasculares Carlos III (CNIC), Madrid, Spain; ^2^Department of Immunology, School of Medicine, Universidad Complutense de Madrid, 12 de Octubre Health Research Institute (imas12), Madrid, Spain

**Keywords:** lectin receptors, signaling, monocytes, macrophages, dendritic cells, innate immunity, inflammation

## Abstract

Myeloid C-type lectin receptors (CLRs) are important sensors of self and non-self that work in concert with other pattern recognition receptors (PRRs). CLRs have been previously classified based on their signaling motifs as activating or inhibitory receptors. However, specific features of the ligand binding process may result in distinct signaling through a single motif, resulting in the triggering of non-canonical pathways. In addition, CLR ligands are frequently exposed in complex structures that simultaneously bind different CLRs and other PRRs, which lead to integration of heterologous signaling among diverse receptors. Herein, we will review how sensing by myeloid CLRs and crosstalk with heterologous receptors is modulated by many factors affecting their signaling and resulting in differential outcomes for immunity and inflammation. Finding common features among those flexible responses initiated by diverse CLR-ligand partners will help to harness CLR function in immunity and inflammation.

## Diversity of Signaling Modules in Myeloid C-Type Lectin Receptors (CLRs)

**Figure 1:**
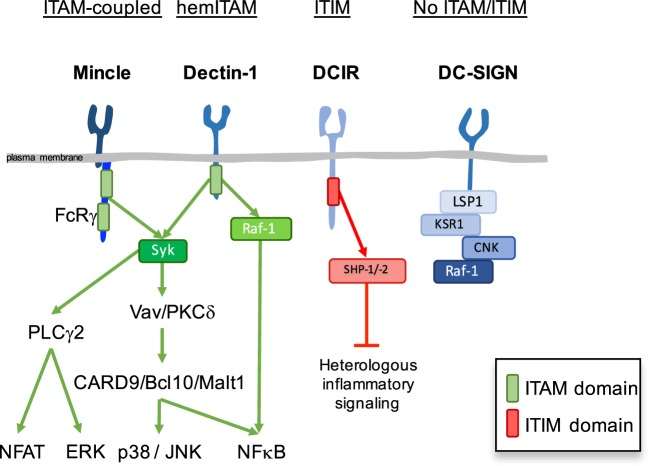
Canonical signaling modules in myeloid C-type lectin receptors (CLRs). Based on canonical intracellular signaling motifs, myeloid CLRs can be classified into immunoreceptor tyrosine-based activating motif (ITAM)-coupled CLRs, hemi-ITAM-(hemITAM)-bearing CLRs, immunoreceptor tyrosine-based inhibitory motif (ITIM)-containing CLRs, and a group of CLRs lacking typical signaling motifs. Mincle, Dectin-1, DCIR, DC-SIGN, and their corresponding canonical signaling pathways and adaptors are depicted as prototypical examples of each category.

The expression of diverse pattern recognition receptors (PRRs), including differential expression of CLRs, provides different subsets of immune cells with a repertoire to interpret and respond distinctly to the information coming from the environment. Myeloid cells are central for initiation and regulation of innate and adaptive immunity or tolerance and the CLR repertoire essentially contributes to myeloid cell function. We previously proposed a classification of myeloid CLRs based on their intracellular signaling motifs (1). While signaling motifs allow to predict effector responses following sensing by CLRs, this canonical response is subjected to modulation by the physical nature, affinity, and avidity of the ligand (2). Based on their intracellular signaling motifs, myeloid CLRs can be classified into the following broad categories (Figure 1): immunoreceptor tyrosine-based activating motif (ITAM)-coupled CLRs, hemi-ITAM-(hemITAM)-bearing CLRs, immunoreceptor tyrosine-based inhibitory motif (ITIM)-containing CLRs, and a group of CLRs lacking typical signaling motifs (1, 3, 4).

Immunoreceptor tyrosine-based activating motif-coupled CLRs have a classical ITAM motifs in their intracellular tail, consisting of YXXL tandem repeats, or can interact with ITAM-containing adaptor proteins, as Fc receptor γ (FcRγ) chain or DNAX-activation protein 12 (DAP12) (5). The majority of them, including Dectin-2 (*CLEC6A* in human, *Clec4n* in the mouse), Mincle (*CLEC4E*), MCL (*CLEC4D*), BDCA-2 (human *CLEC4C*), DCAR (mouse *Clec4b1*), DCAR1 (mouse *Clec4b2*), and mannose receptor (MR) (*MRC1*, CD206) utilize the FcRγ chain adaptor, while MDL-1 (*CLEC5A*) interacts with DAP12 (6–12). Hemi-ITAM-bearing CLRs contain a single tyrosine within an YXXL motif in their cytoplasmic domain (13, 14). Dectin-1 (*CLEC7A*), CLEC-2 (*CLEC1B*), DNGR-1 (*CLEC9A*), and SIGN-R3 (mouse *Cd209d*) belong to the hemITAM-based CLRs category (15–20).

These ITAM or hemITAM CLRs are considered activating receptors that couple to the spleen tyrosine kinase (Syk) (Figure 1) (15, 21, 22). Phosphorylation of the tyrosine(s) in the ITAM or hemITAM motifs generates docking sites for the SH2 domains of Syk, which undergoes a conformational change that permits autophosphorylation and activation (23). Mincle acts as a prototypical activating CLR after recognition of glycolipids in the cell wall of some fungal and bacterial pathogens (24–26). Through the full ITAM of the FcRγ chain adaptor, Mincle couples to Syk and activates Vav proteins and PKCδ, which lead to downstream activation of CARD9/Bcl10/Malt1 and MAPK pathways, thus resulting in the induction of several cytokines and chemokines, including TNF-α, macrophage inflammatory protein 2 (MIP-2; CXCL2), keratinocyte-derived chemokine (KC; CXCL1), and IL-6 (7, 27, 28). Production of inflammatory cytokines by myeloid cells, together with the generation of Th1 and Th17 responses, contribute to protective immunity upon recognition of some Mincle ligands (29–38).

Spleen tyrosine kinase activation downstream of the hemITAM-bearing CLR Dectin-1 leads to similar signaling pathways to those described for Mincle (Figure 1), with activation of the CARD9/Bcl10/Malt-1 module that promotes canonical NF-κB signaling (27, 28, 39). Dectin-1 can also activate MAPK (40, 41), NFAT through phospholipase C-γ2 (42, 43), and a Syk-independent non-canonical NF-κB activation relying on the activation of the Raf-1 kinase (44). These integrated pathways mediate production of reactive oxygen species (ROS) and cytokines, such as IL-1β, IL-6, IL-10, IL-12, TNF-α, and IL-23 to drive Th1 and Th17 differentiation, being essential for the development of antifungal immune responses (45–48). This axis is also activated in response to intestinal fungi, where Dectin-1 contributes to gut homeostasis (49).

Immunoreceptor tyrosine-based inhibitory motif-containing CLRs negatively regulate signaling initiated by kinase-associated heterologous receptors through the recruitment of tyrosine phosphatases, such as Src homology region 2 domain-containing phosphatase (SHP)-1 or -2 (Figure 1). Myeloid CLRs included in this group are human DCIR (*CLEC4A*), mDcir1 (*Clec4a2*), mDcir2 (*Clec4a4*), Clec12a (MICL, DCAL-2, KLRL1, CLL1), MAgH (*CLEC12B*), and Ly49Q (1, 50, 51). The ITIMs of both hDCIR and mDCIR1 have been shown to mediate inhibitory signaling through activation of the phosphatases, SHP-1 and SHP-2 (52–54). Activation of hDCIR on dendritic cells (DCs) leads to inhibition of TLR8-mediated IL-12 and TNF-α production and TLR9-induced IFN-α production (55, 56). Sensing endogenous ligands by DCIR modulates innate immunity to pathogens, such as *Plasmodium* or *Mycobacterium* (57, 58).

Myeloid CLRs that do not bear evident ITAM or ITIM domains include MMR (*MRC1*), DEC-205 (*LY75*), human DC-SIGN (*CD209*), mouse SIGN-R1 (*Cd209b*), Langerin (*CD207*), human MGL (*CLEC10A*), mouse Mgl1 (*Clec10a*), mouse Mgl2 (*Mgl2*), CLEC-1 (*CLEC1A*), human DCAL-1 (*CLECL1*), LOX-1 (*OLR1*), and LSECtin (*CLEC4G*). As an example, DC-SIGN intracellular tail is associated with a signalosome composed of the scaffold proteins LSP1, KSR1, and CNK and the kinase Raf-1 in unstimulated DCs (59) (Figure 1). Similar to other CLRs in this group, DC-SIGN cannot promote DCs activation or cytokine secretion *per se*, but it rather modulates signaling by heterologous receptors (see below) or engages the endocytic machinery contributing to antigen processing and presentation to T cells (3).

Along this review, we will provide illustrative examples of how signaling pathways triggered by a CLR coupled to a particular canonical motif can vary depending on many factors. We will focus on Mincle, Dectin-1, DNGR-1, DCIR, and DC-SIGN as myeloid CLRs representative of each category of signaling motif. Table 1 includes the signaling module coupled to each CLR surveyed in this review, common and gene names, category of flexible signaling source, signaling pathway involved, and the inflammatory outcome provided by such flexibility. In this Table 1, CLRs are grouped based on the signaling module they bear (left column) and graphically illustrates how the signaling pathways triggered by these receptors are more complex and versatile (right columns) than expected by their signaling modules.

**Table 1:** Myeloid C-type lectin receptors (CLRs) surveyed in this review.

Signaling module	Common name	Gene name	Source of flexible signaling	Signaling pathway^a^	Flexibility outcome^b^
No immunoreceptor tyrosine-based activating motif (ITAM) or immunoreceptor tyrosine-based inhibitory motif (ITIM)	DC-SIGN	*CD209*	Homotetramerization	LSP1–KSR1–CNK–Raf-1	Intrinsic (80)
			Sensing self and non-self	LSP1–IKKε–Bcl3	Inhibitory (113–115)
				KSR1–CNK–Raf-1	Activating (59, 110)
			Heterologous modulation	(?)	Activating (148)
				Raf-1–MEK	Inhibitory (112)
			Heterotrimerization DC-SIGN/MR/MDL-1	DNAX-activation protein 12 (DAP12)	Activating (86)

ITAM 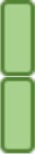	MDL-1	*CLEC5A*	Heterotrimerization DC-SIGN/MR/MDL-1	DAP12	Activating (86)
	Mannose receptor (MR)	*MRC1*	Heterotrimerization DC-SIGN/MR/MDL-1	DAP12	Activating (86)
			Inhibitory ITAM	Fc receptor γ (FcRγ)–Grb2−SHP-1	Inhibitory (12)
	Dectin-2	*CLEC6A, Clec4n*	Heterodimerization Dectin-2/MCL	FcRγ–spleen tyrosine kinase (Syk)–NF-κB p65	Activating (85)
	MCL	*CLEC4D*	Heterodimerization Mincle/MCL	FcRγ–Syk	Activating (81–84)
	Mincle	*CLEC4E*	Heterodimerization Mincle/MCL	FcRγ–Syk	Activating (81–84)
			Inhibitory ITAM	FcRγ–Syk–SHP-1	Inhibitory (91, 92)
			Sensing self	Retarded Syk	Inhibitory (104, 105)
				FcRγ–Syk	Activating (7, 100–103)
			Heterologous modulation	FcRγ−Syk	Activating (140, 141, 143)
				FcRγ−Syk–PKB–Mdm2	Inhibitory (146)

hemITAM 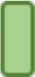	CLEC-2	*CLEC1B*	Homodimerization	Syk	Intrinsic (75, 76)
	DNGR-1	*CLEC9A*	Motif context	Syk	Intrinsic (19, 60–64)
	Dectin-1	*CLEC7A*	Subcellular location	Syk	Intrinsic (67–70)
			Ligand size-conditioned subcellular location	Syk–MAPK–reactive oxygen species	Intrinsic (71–74)
			Phosphatase association	SHP-1–PTEN–FcRγ SHIP-1	Inhibitory (93, 94)
				SHP-2–Syk	Activating (95)
			Heterologous modulation	Syk	Activating (124–126)
				PI3K–mTOR–HIF-1α	Activating (130–134)
				Syk–Pyk2–ERK–SOCS-1	Inhibitory (128)

ITIM 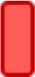	DCIR	*CLEC4A, Clec4a2*	Activating ITIM	IFNI–STAT1 SHP-2 hijacking (?)	Activating (58)
			Sensing self and non-self	(?)	Activating (57)
				SHP-2/SHIP-1	Inhibitory (108, 109)

*^a^Described in the indicated reference. In case it was not studied in depth, it might be incomplete*.

*^b^This column indicates the inflammatory balance provided by each source of signaling flexibility. “Intrinsic” refers to specific responses triggered by particular CLRs*.

## Signaling Flexibility Beyond the Canonical Motifs

### Motif Context and Receptor Location Modulate Signaling

**Figure 2:**
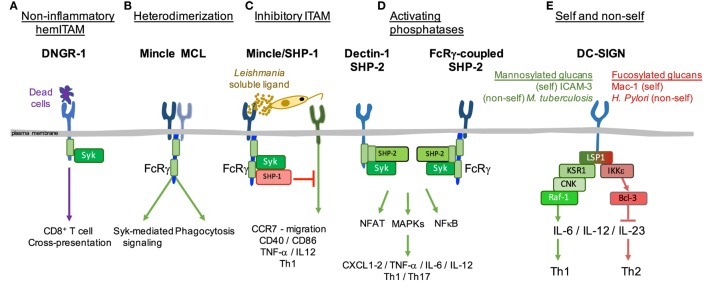
Signaling flexibility downstream of C-type lectin receptors (CLRs). Signaling triggered downstream of CLRs goes beyond the canonical modules present in their intracellular domains and can be modulated by different processes. Some examples of such plasticity are represented. **(A)** DNGR-1 promotes cross-presentation of antigens to CD8^+^ T cells, yet not directly contributing to inflammation. **(B)** Mincle and MCL dimerize, boosting phagocytosis, and spleen tyrosine kinase (Syk)-mediated inflammatory responses. **(C)** Sensing of a soluble ligand from *Leishmania* by Mincle triggers an inhibitory immunoreceptor tyrosine-based activating motif conformation downstream of Fc receptor γ (FcRγ), where SHP-1 dampens inflammatory responses triggered by heterologous receptors. **(D)** The phosphatase SHP-2 acts as a scaffold downstream of Dectin-1 and FcRγ-coupled CLRs, facilitating the recruitment of Syk and its inflammatory signaling. **(E)** Both self and non-self ligands share signaling pathways downstream of DC-SIGN depending on whether they are mannosylated or fucosylated glucans.

Classifications of receptors based on intracellular structural motifs stand on the fact that those domains determine the molecular signaling pathways initiated after ligand recognition (1). However, in addition to the basic ITAM and ITIM motifs, subtle variations in the context of the canonical motifs profoundly affect the signal delivered. For example, DNGR-1 is a DC-specific hemITAM-bearing receptor that detects dead cells and promotes cross-presentation in sterile or infectious settings, without contributing to inflammation (Figure 2A), in contrast to the close-related Dectin-1 (19, 60–63). This deficiency to promote cytokine production through DNGR-1 hemITAM was linked to an isoleucine that precedes the tyrosine in DNGR-1 hemITAM and rescued by mutation to the glycine present in Dectin-1 hemITAM (60). Signaling flexibility can thus be intrinsically provided by the amino acid sequence of those motifs present in a CLR. In this regard, residues in the neck region of DNGR-1 allow the receptor to adopt different conformations that depend on pH and ionic strength, modulating its function as the receptor progresses through the endocytic pathway (64). Even the inflammatory response of mouse and human Dectin-1 to the same ligand varies because of minor interspecies variations in the signaling motif, with low valency ligands inducing proinflammatory genes through human but not mouse Dectin-1 (65).

Receptor location also affects CLR signaling and functions. A single CLR may be expressed in different cell types (66) as diverse isoforms that may differ in subcellular location. For example, two isoforms of Dectin-1 have been described to bind β-glucans (67); isoform A is characterized by the presence of a stalk region including an N-linked glycosylation site, which is missing in isoform B (68). This glycosylation determines the cell surface expression of isoform A, while non-glycosylated isoform B is retained intracellularly, thus conditioning the response to ligands (69) and the sensitivity to proteolytic cleavage (70).

The subcellular location of a CLR may not only depend on intrinsic features in its sequence, but also on the size of the particle where the ligand is recognized. For example, “frustrated” phagocytosis mediated by Dectin-1 in response to ligands exposed in large particles leads to enhanced cytokine response and ROS production compared with soluble ligands (71–73). Blockade of Dectin-1 internalization following ligand exposure leads to sustained MAPK activation (72), suggesting that endocytosis dampens Dectin-1 production of cytokines. Thus, formation of a phagocytic synapse by particulate β-glucan redistributes Dectin-1 and phosphatases along the cellular membrane, favoring proinflammatory signals including ROS production (73). In addition, the size of the ligand-containing particle and the consequent location of the receptor, can lead to qualitatively different responses. Dectin-1-mediated phagocytosis dampens the nuclear translocation of neutrophil elastase, controlling the extent of neutrophil extracellular traps (NET) formation in response to small pathogens (bacteria or yeast). Consequently, Dectin-1 blockade or deficiency leads to enhanced NETosis, as observed in response to non-phagocytic large pathogens (hyphae) (74).

Thus, the expected canonical response based on signaling modules can be altered both by slight modifications in motif context and the subcellular location of CLRs, taking into account that the latter may be affected by the size of the ligand recognized.

### Multimerization of CLRs for Signaling

The signal transduction through several myeloid CLRs may also depend on their capacity to form dimers or multimers with other CLRs. CLRs bearing hemITAMs may require two phosphorylated tyrosines in a homodimer to bind Syk. It has been shown that CLEC-2 preexists as a dimer that aggregates following ligand binding (75, 76). The hemITAM motif of CLEC-2 is crucial for blood-lymph separation during development (77, 78). Of note, thrombus stability is dependent on CLEC-2 but not on the hemITAM, revealing a hemITAM-independent signaling for CLEC-2 (79).

DC-SIGN provides another example of homomultimerization, despite lacking ITAM or ITIM domains. This CLR appears assembled as a tetramer, allowing multiple interactions with diverse pathogens that differ in size, but also increasing ligand avidity (80). In addition, some CLRs form heterodimers, such as MCL and Mincle (11, 81). These two CLRs are interrelated as they both sense the mycobacterial glycolipid trehalose-6,6-dimycolate (TDM), triggering an FcRγ-dependent pathway (11). Indeed, MCL and Mincle are co-regulated and depend on each other for their mutual surface expression (82, 83). However, the association of MCL with FcRγ in this complex is species-specific, being direct in mouse cells (11) but requiring Mincle in rat (81). Thus, the interaction between these CLRs would facilitate MCL signaling capacity *via* association with Mincle and translocation to the plasma membrane. On the other hand, Mincle would benefit the endocytic capacity of MCL (Figure 2B) and both receptors could increase affinity or specificity for their ligands (84). MCL also forms a heterodimeric pattern-recognition receptor with Dectin-2 (85), which has a high affinity for α-mannans on the surface of *Candida albicans* (*C. albicans)* hyphae.

Cooperative interaction is also found in the case of dengue virus binding with high affinity to MR and DC-SIGN, receptors that subsequently handle the virus to the lower affinity receptor CLEC5A, which mediates signal transduction (86).

All these examples illustrate how multimerization of CLRs, forming either homo- or hetero-complexes, facilitates a cooperative response to the ligand.

### Is the Function of CLRs Inhibitory or Activating?

Another layer of complexity in CLR signaling stems from the ability of a single CLR to bind different ligands through its plastic C-type lectin domain. For instance, depending on their relative affinity or avidity, ligands may fine-tune signaling pathways downstream of ITAM motifs. Whereas the binding of high-avidity ligands to these receptors induces activating signals, the binding of low-avidity ligands leads to hypophosphorylation of the ITAM domain and preferential association of SH2-containing phosphatases like SHP-1, a configuration known as “inhibitory ITAM” (87). Although FcαRI receptor, which associates for signaling with the FcRγ chain, is the paradigmatic example of this inhibitory pathway (88–90), we have shown that CLRs associated with FcRγ chain may behave in the same fashion.

As an example, Mincle senses a soluble ligand derived from *Leishmania* that induces phosphorylation of SHP-1 coupled to FcRγ chain, inhibiting DC activation through heterologous receptors (Figure 2C) (91). In addition, SHP-1 contributes to deceleration of phagosome maturation upon TDM binding, suggesting an inhibitory signal downstream of Mincle during phagocytic processes (92). MR binds the FcRγ chain and, upon sensing *Mycobacterium tuberculosis*, recruits SHP-1 to the phagosome, thus limiting PI(3)P generation and delaying fusion with the lysosome, which promotes *M. tuberculosis* growth (12). Following treatment of DCs with curdlan or depleted zymosan (lacking TLR-stimulating properties), Dectin-1 signaling is modulated by the association of SHP-1 and PTEN to the FcRγ chain, hindering cytokine expression, DC maturation, and T-cell proliferation (93). ROS production downstream of Dectin-1 sensing of *C. albicans* is also tightly regulated by the SH2-domain containing inositol 5′ phosphatase (SHIP)-1 in response to Dectin-1 ligands (94). Thus, association of phosphatases to “activating” CLRs depending on the ligand nature, binding affinity, or avidity may contribute to maintenance of immune homeostasis.

Conversely, tyrosine phosphatases can contribute to activation. Contrary to SHP-1, the related tyrosine phosphatase SHP-2 acts as a scaffold, facilitating the recruitment of Syk to Dectin-1 or the adaptor FcRγ chain (95) (Figure 2D). In this way, DC-derived SHP-2 was crucial *in vivo* for the induction of TNF-α, IL-6, IL-12, and Th1 and Th17 anti-fungal responses upon *C. albicans* infection (95).

Immunoreceptor tyrosine-based inhibitory motif-coupled receptors can also deliver an activating signal. In a model of tuberculosis infection in non-human primates, DCIR deficiency impairs STAT1-mediated type I IFN signaling in DCs, leading to increased production of IL-12 and differentiation of T lymphocytes toward Th1. Thus, DCIR-deficient mice with increased Th1 immunity control *M. tuberculosis* better than WT animals, but also shown increased inflammation in the lungs mediated by TNF-α and inducible nitric oxide synthase (iNOS) (58). This study suggests that DCIR acts as an activating receptor for the STAT1-type I IFN signaling, and speculates that DCIR may function as a molecular sink binding unphosphorylated inactive SHP-2, therefore, limiting SHP-2′s capacity to deactivate STAT1.

The examples explained above illustrate a lack of correspondence between the canonical motif coupled to a CLR and the resulting signaling pathway. Association to kinases would lead to activating routes, while association to phosphatases would result in regulatory pathways, with some exceptions like the SHP-2-mediated CLR-induced activation (95). Association of kinases or phosphatases could be related to the strength of the initiating signal, with suboptimal phosphorylation leading to phosphatase binding to the hypo-phosporylated ITAM (inhibitory ITAM) (87). Due to the signaling flexibility offered by CLRs, a detailed empiric analysis for each CLR-ligand interaction in terms of type of ligand, concentration, and kinetics of exposition would be required to predict the signaling outcome.

### Dealing With Self and Non-Self

C-type lectin receptors act as plastic receptors, some of them detecting self-ligands, other detecting non-self ligands, and many of them acting as dual receptors sensing self and non-self. It is possible that CLRs will behave as activating receptors when they sense non-self ligands, while CLRs bearing an ITIM motif will preferably bind self to dampen inflammation. However, in opposition to non-dangerous self, also known as “self-associated molecular patterns” (96, 97), Polly Matzinger proposed the existence of dangerous-self (damage-associated molecular patterns or DAMPs) exposed and/or released upon necrotic cell death (98, 99). In addition, tissue damage signals concomitant to infection can contribute to effector responses. Thus, DNGR-1 senses tissue damage concomitant with viral infections and facilitates antigen processing of viral antigens for cross-presentation to CD8^+^ T cells, decoding the antigenicity rather than the adjuvanticity of the cargo (60–63). Some examples of CLRs dealing with self and non-self ligands are explained below.

Mincle is a plastic CLR promoting proinflammatory signals after sensing glycolipids in the cell wall of bacteria and fungi (24–26), but also sensing damaged self in the form of soluble SAP-130 following necrosis (7). Mincle sensing of β-glucosylceramide (100) or cholesterol sulfate (101) promotes immunopathology (102, 103). Conversely, there are reports suggesting that Mincle sensing of SAP-130 can also drive immunosuppression (104). Moreover, human albumin abolishes innate immunity by directly binding Mincle receptor in the microglia after subarachnoid hemorrhage (105). Thus, Mincle is an example of CLR that deals with self and non-self ligands that may result in activating or inhibitory signals. However, the correlation of sensing self with an inhibitory response and sensing non-self with an activating response is not established. In this regard, non-self signals from pathogens may mimic self-inhibitory signals to escape immune surveillance, which could be the case for Mincle sensing of *Leishmania* (91).

DCIR is a myeloid CLR endowed with an ITIM motif that behaves as a self PRR. DCIR maintains the homeostasis of the immune system (106), since aged mice deficient for this CLR spontaneously develop several autoimmune disorders (107). Intravenous immunoglobulins bearing sialic acid induce a DCIR-mediated negative signal in DCs *via* SHP-2 and SHIP-1 that promotes Treg differentiation and dampens allergy (108). DCIR self-sensing can also occur in the context of infection, thus modulating the inflammatory response. DCIR-deficient mice exhibited severe inflammatory disease following Chikungunya virus infection (109). However, reduced adaptive T-cell responses in DCIR-deficient mice following cerebral malaria caused by *Plasmodium berghei* renders them more resistant (57). Since no evidence for direct interactions between DCIR and Chikungunya virus and *P. berghei* exists, we could hypothesize that DCIR may be recognizing DAMPs released during infection.

DC-SIGN illustrates how a single CLR deals differently with a variety of self and non-self ligands. DC-SIGN binds high mannose and fucose (LeX, LeY, LeA, LeB) that can be exposed in a variety of self receptors, such as ICAM-2, ICAM-3, CEACAM-1, Mac1 and CEA, or non-self proteins (structures in pathogens, including viruses, bacteria, fungi, and eukaryote parasites) (3, 110–115). Upon binding of mannosylated glucans, either self as those present on ICAM-3 (110) or non-self from *M. tuberculosis* (59), DC-SIGN couples to a LSP1–KSR1–CNK signalosome, leading to activation of Raf-1 and acetylation of the NF-κB p65 subunit, which results in enhancement of proinflammatory responses, including IL-12p70 and IL-6, although also promotes IL-10 transcription (59) (Figure 2E). In contrast, DC-SIGN recognition of fucosylated glucans as presented in self proteins, such as Mac1 (113) or non-self pathogens (*Helicobacter pylori*) (114), leads to dissociation of the LSP1-based signalosome and leaves just LSP1 associated with DC-SIGN. Phosphorylated LSP1 subsequently recruits IKKε and CYLD. IKKε activation inhibits CYLD deubiquitinase activity, facilitating nuclear translocation of ubiquitinated Bcl3 that represses TLR-induced proinflammatory cytokine expression, enhancing expression of IL-10 and Th2-attracting chemokines, and thus promoting Th2 polarization (114) (Figure 2E). In addition, IKKε collaborates with type I IFNR signaling to induce and activate the transcription factor ISGF3 that induces IL-27p28, a key cytokine for induction of T follicular helper cells (115). These results point to DC-SIGN as a dual receptor that, depending on the nature of the ligand, contributes to maintain homeostasis or initiates the immune response against some pathogens.

All these examples illustrate how a single CLR can trigger different signaling pathways depending on the recognition of self or non-self ligands. Current understanding of these processes is based on the study of individual CLRs. Deciphering common signaling patterns for self versus non-self sensing would allow harnessing immunity and inflammation by CLRs.

## Modulation of Heterologous Signaling by Myeloid CLRs

In addition to the diverse response of a single CLR depending on the stimulus, it is fascinating how these signaling pathways interact with signals from heterologous receptors and lead to complex responses to stimuli that are simultaneously detected by several myeloid PRRs expressed in myeloid cells [see also Ref. (116, 117) for reviews focused on this topic]. In this section, we illustrate some examples of how myeloid CLRs cross-talk with surrounding heterologous receptors.

### Dectin-1 Affects Simultaneous and Deferred Signaling Through Heterologous Receptors

**Figure 3:**
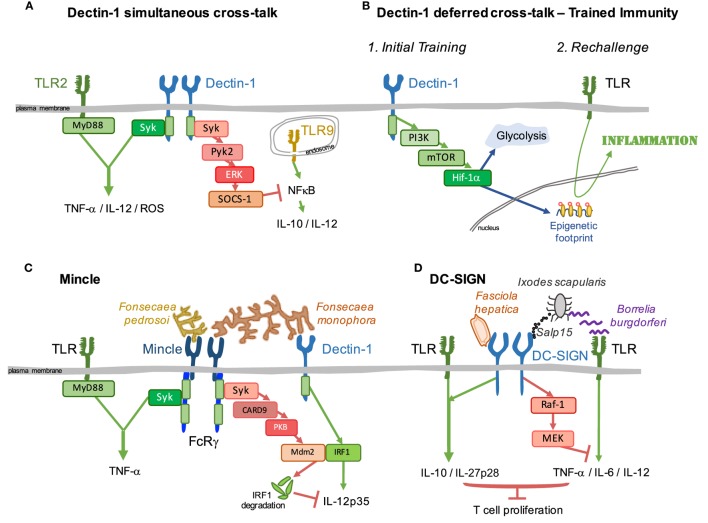
Signaling crosstalk between myeloid C-type lectins and heterologous receptors. Signaling pathways initiated downstream of C-type lectin receptors (CLRs) interact with surrounding cascades triggered by heterologous receptors. Examples of such crosstalk are illustrated. **(A)** Dectin-1 coordinates with simultaneous signals from diverse TLRs to modulate the inflammatory response; this interaction can be either positive, as for TLR2/MyD88 (left), or negative, as for TLR9 through a Pyk2/ERK/SOCS-1-dependent pathway (right). **(B)** In addition, the axis Dectin-1/PI3K/mTOR/Hif-1α generates a switch toward glycolytic metabolism together with an epigenetic footprint, allowing for a “deferred” improved response to TLRs, boosting the inflammatory response. This process is known as trained immunity. **(C)** A full inflammatory response against *Fonsecaea pedrosoi* is achieved by synergistic stimulation between Mincle and ligands for TLRs coupled to the MyD88 adaptor (left). However, simultaneous recognition of *Fonsecaea monophora* by Mincle and Dectin-1 triggers a Mincle-PKB-Mdm2-dependent degradation of Dectin-1-activated IRF1, dampening the expression of protective IL-12p35 (right). **(D)** DC-SIGN recognition of *Fasciola hepatica* enhances TLR-induced IL-10 and IL-27p28 (left). Moreover, DC-SIGN sensing of the salivary protein Salp15 from the tick vector *Ixodes scapularis* dampens inflammatory responses triggered by *Borrelia burgdorferi* through TLRs (right). Both examples illustrate strategies to escape immune surveillance based on inhibition of T cell proliferation.

Dectin-1 triggers a response after sensing infectious agents, such as diverse fungi and mycobacteria (118), *Salmonella typhimurium* (119) or *Leishmania infantum* (120). Dectin-1 may also promote proinflammatory signals following the detection of endogenous factors, such as vimentin from atherosclerotic plaques (121), galectin-9 from pancreatic carcinoma (122), or N-glucans on tumor cells (123). In addition to a prototypical activating CLR, Dectin-1 modulates signals simultaneously triggered through other PRRs. Dectin-1 cooperates with signals from TLR2/MyD88 to increase proinflammatory cytokine production (124–126). This synergy is exerted at the level of effector responses resulting in increased production of TNF-α, IL-12, and ROS (124) (Figure 3A, left). Dectin-1 also positively cooperates in the full activation of the NLRP3 inflammasome, participating in the priming and generation of pro-IL-1β and the induction of ROS required for NLRP3 activation (127). Conversely, Dectin-1 stimulation with depleted zymosan in bone marrow macrophages leads to Syk and Pyk2-ERK-dependent activation of SOCS-1 that downregulates IL-10 and IL-12p40 production induced by TLR9 stimulation (128) (Figure 3A, right). This effect would contribute to the Dectin-1 signature in priming Th17 responses (40, 128). In addition, Dectin-1 protects against chronic liver disease by suppressing TLR4 signaling. This effect is mediated by reducing TLR4 and CD14 expression, which are regulated by Dectin-1-dependent macrophage colony stimulating factor expression (129).

Apart from direct modulation of signaling pathways triggered simultaneously, Dectin-1 can leave a footprint that affects deferred signaling by heterologous receptors, a process named as trained immunity (130). Trained immunity after sensing of *C. albicans* or purified β-glucan *via* Dectin-1 results in enhanced protection to a lethal challenge with *Candida* and cross-protection to *Staphylococcus aureus* infection (130, 131). This increased protection upon a later infection is linked to increased proinflammatory responses to delayed rechallenge with different TLR ligands, such as LPS or Pam_3_Cys_4_ (130) (Figure 3B), or bacteria, i.e., *Bacteroides fragilis, Escherichia coli, Staphylococcus aureus, Borrelia burgdorferi*, or *M. tuberculosis* (130, 132, 133). In monocytes, Dectin-1 signaling triggers the PI3K-Akt pathway, leading to activation of mTOR and HIF-1α (131). This leads to a shift from oxidative phosphorylation to aerobic glycolysis. Accumulation of fumarate, associated with glutamine replenishment of the TCA cycle, inhibits KDM5 histone demethylases, a key step for induction of monocyte epigenetic reprogramming that underlies the long-lasting effects of trained immunity (130, 134) (Figure 3B).

Apart from β-glucan or *Candida*, several other self and non-self ligands, such as chitin (135), BCG vaccine (136), and uric acid (137) induce trained immunity (137, 138). It would thus not be surprising that more CLRs could contribute to trained immunity. In this regard, although *C. albicans* mannans, potentially sensed by MR, Dectin-2, or Mincle (46), have shown not to prime human monocytes directly (130), they are essential for *C. albicans*-induced training (133). Furthermore, both Dectin-1 and MR are needed to trigger glycolysis upon *C. albicans* stimulation (139); this glycolytic switch constitutes a critical metabolic step in trained immunity induction (131, 139). Trained immunity triggered by Dectin-1 and potentially other CLRs is thus a consequence of metabolic switch and epigenetic programming that affects deferred heterologous signaling.

### Mincle-Triggered Regulatory Responses

As described before, Mincle triggers an FcRγ-mediated activating signal in response to different stimuli. In addition, Mincle engagement can deliver regulatory responses affecting signaling pathways triggered by heterologous PRRs, such as TLRs or other CLRs, for example, Dectin-1. This section will explore modulation of heterologous receptors by Mincle.

Mincle is induced following TLR activation (7). Following sensing of *Fonsecaea pedrosoi*, Mincle triggers an incomplete inflammatory response that requires synergistic TLR stimulation to induce a potent proinflammatory response (Figure 3C, left), needed to clear the infection in a mouse model of chromoblastomycosis (140). This cooperative activation through Mincle and TLRs is particularly effective in human newborn DCs. Co-stimulation using the Mincle agonist trehalose-6,6-dibehenate and the TLR7/8 agonist R848 led to enhanced caspase-1 and NF-κB activation, Th1 polarizing cytokine production and autologous Th1 polarization (141).

However, Mincle exhibits a dual role in promotion and subsequent resolution of inflammation. Mycobacteria express ligands for TLRs which induce expression of Mincle that can then detect TDM and contribute to inflammation. Mincle *via* the Syk/p38 axis can also lead to eIF5A hypusination that increases translation efficiency of iNOS, which is transcriptionally induced by TLR2 ligation (142). In this way, Mincle favors NO production that inhibits late-stage activation of NLRP3 inflammasome in TDM-induced inflammation, contributing to termination (142). Similarly, TLR2 sensing of *Corynebacterium* induces robust Mincle expression, which cooperatively detects corynebacterial glycolipids favoring production of granulocyte colony stimulating factor and NO (143).

Dectin-1 and Mincle are involved in the recognition of *Fonsecaea monophora*, a pleomorphic fungus also responsible for chromoblastomycosis (144, 145). Signaling triggered by Dectin-1 initiates protective immunity against the fungus by activating IRF1 and IL-12p35 transcription. However, these responses are dampened by the Mincle/Syk axis, in a process involving PI3K/PKB-mediated activation of the E3 ubiquitin ligase Mdm2, leading to degradation of IRF1 and repression of IL-12p35 production (Figure 3C, right). In this way, Mincle sensing of *F. monophora* dampens induction of protective Th1 immunity triggered by Dectin-1 (146). Mincle is also targeted by *Leishmania* parasites to evade the priming of Th1 immunity initiated by DCs. As explained above, Mincle recruits SHP-1 to an inhibitory ITAM configuration in the coupled FcRγ chain, and this results in inhibition of DC activation by heterologous receptors sensing *Leishmania* or LPS (91) (Figure 2C). Mincle ligation can also reduce TLR4-mediated inflammation, whereas Mincle deletion or knockdown results in exaggerated inflammation in response to LPS. This effect is mediated through the control of TLR4 correceptor CD14 expression (147).

### Tailoring Immunity Through DC-SIGN

DC-SIGN engagement does not generally induce the expression of cytokines by itself, but rather modulates responses initiated by TLRs. Thus, glycans from the helminth *Fasciola hepatica* are recognized by DC-SIGN leading to enhanced TLR-induced IL-10 and IL-27p28, triggering a tolerogenic program that differentiates naive CD4^+^ T cells into regulatory T cells (148) (Figure 3D, left). However, the interaction of DC-SIGN with the salivary protein Salp15 from the tick *Ixodes scapularis* dampens inflammatory responses triggered by *Borrelia burgdorferi*. Raf-1 activation downstream of DC-SIGN sensing Salp15 results in MEK-dependent decrease of IL-6 and TNF mRNA stability and impaired nucleosome remodeling at the IL-12p35 promoter, modulating TLR-induced DC activation and T cell proliferation (112) (Figure 3D, right).

All these examples clearly illustrate how signaling pathways triggered by CLRs can have an impact on responses mediated by surrounding heterologous receptors, adding an extra layer of complexity to our understanding of CLR-mediated responses.

## Concluding Remarks

Classical sorting of myeloid CLRs based on the structure of the C-type lectin domain does not have functional significance. A more recent classification based on the presence of ITAM, hemITAM, or ITIM intracellular signaling motifs associated with the receptors has been useful as a starting point to predict the functional outcome of signaling CLRs (1). However, many factors may alter the expected canonical response. Minor variations in the context of the canonical motifs result in different signaling and effector outcomes (60, 65). Subcellular location depending on the isoform (69) or conformation of the receptor based on specific residues (64) also affects the function of the receptor. CLR signaling also depends on the size of the particle, where the ligand is recognized, affecting quantitatively the strength of the reaction (71–73) and also leading to qualitatively different responses (74, 149). Cooperative binding and signal transduction may be a consequence of multimerization. There are examples of homodimerization (75, 76) and formation of hetero-complexes (11, 81, 84–86). Hetero-complexes result in a mutual benefit for involved receptors, combining avidity for the ligand, capacity for endocytosis and/or signal transduction capabilities.

The plasticity of the C-type lectin domain allows binding to different ligands that, depending on their relative affinity or avidity, may trigger activating or inhibitory signaling pathways downstream of the same motifs. For example, low-avidity ligands drive a Syk-dependent association with SHP-1 to the ITAM domain (87, 88, 90), with a growing list of examples illustrating CLRs coupled to the FcRγ chain (12, 91–93). Conversely, tyrosine phosphatases may contribute to activation (95) and ITIM-containing CLRs may trigger activating signals (58). These results evidence the fine regulation of signaling though a single receptor based on differential interaction with diverse ligands, leading to the hypothesis that sensing self-ligands through CLRs could drive tolerance while non-self ligands could provoke immunity. However, dangerous-self could rather contribute to immunity and some non-self ligands could inhibit immune response for evasion, making the final outcome of a single response rather unpredictable. In addition, the concerted sensing of complex ligands by a variety of PRRs leads to complex integrated responses. CLRs may affect signals of heterologous receptors that are simultaneously triggered, either enhancing or modulating the response (59, 91, 115, 124–126, 128, 142, 146). Of note, Dectin-1 induces a metabolic switch and epigenetic programming that affects deferred heterologous signaling (130, 131). In conclusion, understanding how different signaling pathways triggered by CLRs and heterologous receptors act in concert during sensing self and non-self remain a fascinating endeavor.

Research in the field of CLRs has gained much attention considering the diversity of members, ligands, expression pattern on clinically relevant cellular populations and their relevant function on the initiation, and regulation of immunity and inflammation. Some of these features have been illustrated here and offer multiple possibilities to harness CLR-triggered responses. However, CLR manipulation may lead to unexpected outcomes and needs to be tested empirically. In addition, deciphering molecular signatures common to signaling pathways triggered by CLRs in response to different ligands will help to understand their precise role in immunity and inflammation.

## Author Contributions

CF, SI, PS-L, MM-L, and DS conceived and wrote the manuscript. CF did the figures that were edited by all the authors.

## Conflict of Interest Statement

The authors declare that the research was conducted in the absence of any commercial or financial relationships that could be construed as a potential conflict of interest.
